# Possible role of Sox11 in a rat model of surgical brain injury

**DOI:** 10.22038/IJBMS.2024.71455.15537

**Published:** 2024

**Authors:** Jiafeng Tang, Muyao Wu, Jinchao Shen, Lei Jiang, Lifen Chen, Baoqi Dang

**Affiliations:** 1 Department of Rehabilitation, Zhangjiagang TCM Hospital Affiliated to Nanjing University of Chinese Medicine, Zhangjiagang, China; 2 Department of Anesthesiology, Zhangjiagang TCM Hospital Affiliated to Nanjing University of Chinese Medicine, Zhangjiagang, China; # These authors contributed equally to this work

**Keywords:** Apoptosis, Brain edema, Brain injury, Necrosis, Neurosurgery, Sox11

## Abstract

**Objective(s):**

Sox11, one of the SoxC family members, is an important transcription factor during neural development and neurogenesis. However, there is no report about its function in neural apoptosis. This research aims to examine the function of Sox11 in surgical brain injury (SBI).

**Materials and Methods:**

We used 90 Sprague-Dawley rats to develop the SBI models and the siRNA of Sox11 to study the roles of Sox11. Western blot, real-time PCR, immunofluorescence, neuron apoptosis and necrosis, brain edema, and neurological score were determined.

**Results:**

The gene and protein amount of Sox11, compared with the Sham group, were increased after SBI, which reached a peak at 12 hr. In addition, following the application of siRNAs, the amount of Sox11 protein was significantly less than that in the SBI group. On the other hand, neuronal apoptosis, necrosis, and brain edema were significantly increased, while neurological scores were decreased.

**Conclusion:**

These findings demonstrate the role of Sox11 following nerve injury induced by SBI. Inhibition of Sox11 with siRNA may lead to neuronal injury and cell death, aggravating secondary brain injury after SBI.

## Introduction

When conducting neurosurgery, incision, retractor stretch, and electrocauterization brain cells around the surgical resection region may mistakenly be harmed, this is called surgical brain injury (SBI)(1). Although it is recognized as an important clinical problem, effective treatments have not yet been developed (2). SBI destroys the balance of the brain environment, which causes pathophysiological complications, including brain edema, inflammation of nerve tissue, loss of nerve function, and neuron and glial cell death (3, 4). To effectively prevent and treat early brain injury, drugs can be used to reduce the disability rate and mortality of SBI.

The sex-determining regions-Y box (SOX) gene family is made up of many supergenes with high-motility-group (HMG) box-conserved motifs and is identified as an important transcription regulator. The SOX gene family has been subdivided into eight subfamilies A-H (5, 6). Members of the same group share similarities within and outside the HMG box domain, whereas elements of different cohorts are less similar. Sox11, as well as Sox4 and Sox12, are elements of Group C (7). Studies have shown that developing mouse embryos have a wide expression of Sox11, especially in neurons and mesenchymal tissues (8, 9). The protein has revealed multiple particular functions, such as regulating epithelial-mesenchymal interactions, kidney and lung development, neuronal determination and differentiation, inductive tissue remodeling, axonal growth, and sensory neuron survival (10-12). *In vitro* evidence is recently emerging that the Sox11 expression in cerebral cortex nerve cells is controlled by neuronal depolarization, while electroconvulsive stimulation can also regulate Sox11 in adult rats *in vivo* (13). These findings revealed that the expression of Sox11 is controlled by the neuronal activity in the brain. Furthermore, some studies have proved that Sox11 can modulate some functions in adult neurons such as pathological and physiological triggering. Axotomy and low oxygen levels can induce Sox11 expression in mature central and peripheral neurons, which indicates that Sox11 is an integral part of the neuronal injury response (14). Sox11 can be highly elevated after temporary forebrain ischemia in the cerebral cortex, piriform cortex, amygdala, and hippocampal formation in adults, further indicating that it may have a function in controlling the pathogenic effects of brain damage (15). However, the specific molecular mechanisms of Sox11 function remain unclear and thus need further study.

In summary, evidence has shown that Sox11 is expressed in brain injury. However, the effect of Sox11 after SBI is not identified. Hence, this research aimed to evaluate Sox11 expression in a rat model of SBI as well as potential Sox11 post-SBI strategies.

## Materials and Methods

***Study design and experimental groups***

**Figure 1:**
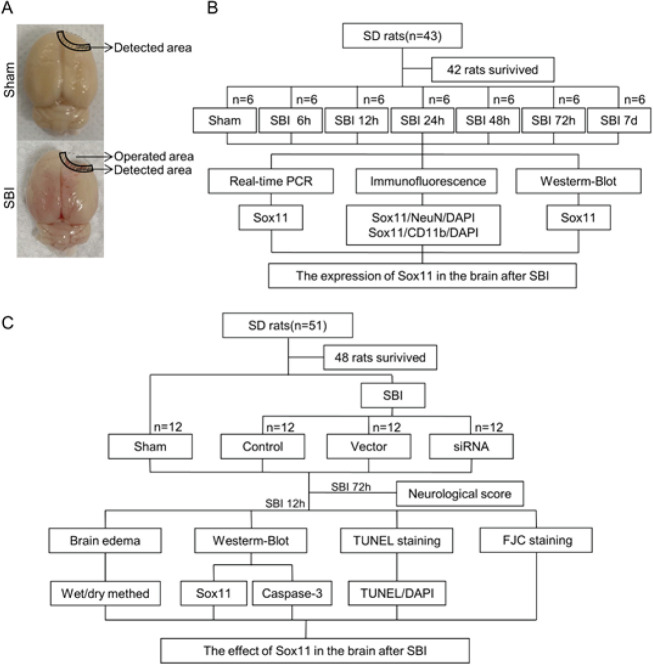
Experimental design

Experiment 1: The rats used had the same body mass, feed inputs, and motor skills. To monitor and follow the time course of Sox11 after SBI, the 42 rats (42 staying alive out of 43) were divided into seven random cohorts based on digital randomization (EXCEL randbetween function), particularly Sham, SBI 6 hr, SBI 12 hr, SBI 24 hr, SBI 48 hr, SBI 72 hr, and SBI 7 d. Specimens of the brain tissue around the surgical site were collected. One group was selected to carry out a western blot (WB) evaluation to examine the expression of Sox11 in SBI rat brains. At the same time, the other one was chosen to carry out real-time PCR (RT-PCR). In addition, the Sox11 cellular localization was characterized by double immunofluorescence analysis at 12 hr following SBI (Figure 1B).

Experiment 2: To detect the importance of Sox11 in SBI, 48 rats (48 remaining out of a cohort of 51) were randomly divided into four cohorts determined with EXCEL and randbetween function as follows: Sham, Control, Vector, and siRNA. At 12 hr after SBI (time established after Experiment 1), the brain tissue around the operation zone was extracted for WB to define the expression of Sox11, brain water content, Fluoro-Jade C (FJC) staining, and terminal deoxynucleotidyl transferase-mediated dUTP nick-end labeling (TUNEL) staining to assess the neuronal apoptosis and necrosis. In addition, a neurological test was carried out at 72 hr following SBI (Figure 1C). The blind method was applied to the experiment. In brief, an independent researcher coded each specimen so that all specimens were unknown to all investigators.

***Animals***

The Animal Ethics and Welfare Committee (AEWC) of Zhangjiagang TCM Hospital Affiliated with Nanjing University of Chinese Medicine authorized the research proposal, and all animal approaches were done after the Chinese Association for Laboratory Animal Sciences (16). Male Sprague-Dawley rats weighing 350-400 g, acquired from the Animal Center of Soochow University in Suzhou, China, were employed in this research. The rats were housed in a 12:12 hr light: without light round while being provided access to eat and drink at will.

***SBI rat model***

Partial removal of the right frontal lobe in the rat was used to create the SBI model (17). In brief, animals received intraperitoneal anesthesia with 4% sodium pentobarbital (40 mg/kg) and then kept in the stereotactic instrument. Next, a midline scalp cut was performed and a 5 mm ×5 mm square cranial window, achieved with a micro drill, was located in the right frontal bone, along the sagittal and coronal planes, 2 mm lateral and 1 mm anterior to the bregma. To expose the right frontal lobe, the dura was incised and reflected. We used a blade to excise the exposed tissue. After successfully achieving hemostasis, the skin was sutured. All rats were monitored during post-operative recovery in a comfortable environment and returned to their home cages. Sham rats were subjected to the same procedures, including only the craniectomy procedure.

***Drug injection***

We administered siRNA via intracerebroventricular injection to rats to examine if the altered expression of Sox11 was associated with SBI-induced brain damage (18-20). After anaesthetization with 4% sodium pentobarbital intraperitoneally, animals were first placed in a stereotactic device. A 10 µl Hamilton syringe was employed to inject the medicines into the right lateral ventricles via a burr opening (Hamilton Company, United States). The directions to the bregma were 1.5 mm backward, 1 mm laterally, and 3.2 mm down to the horizontal level of the skull. Then, Sox11 siRNA (SO-2803412G, 500 pmol, Dharmacon Inc, United States) was delivered at 0.5 µl/min via the burr opening. The siRNA sequence was GAGUUAAAGUGAAAUGAGU. The syringe was preserved in place after the administration for 10 min to avoid leaking, then gently removed. Bone wax was employed to fill the burr opening, and the skin cut was repaired. The recovery of the rats was carefully monitored.

***Tissue collection and sectioning***

Rats were sedated with 4% pentobarbital sodium after injury at set intervals. To perform molecular analysis, the rats were given 200 ml of 0.9% normal saline at 4 ^°^C by transcardial perfusion. Cortical samples <3 mm from the edge of the injured area were collected and placed on ice and then kept at -80 ^°^C until further analysis. The same method was used in the Sham group (Figure 1A). 

The brain sections collected were immediately soaked in 4% paraformaldehyde overnight and inserted in paraffin, and 5 µm width sections were cut for the examinations. The processes were carried out by two researchers who were unaware of the treatments.

***Real-Time PCR***

Total RNA extraction was performed from distal areas of injured brain tissue utilizing the Trizol reagent (Invitrogen, Carlsbad, CA, United States), and 1 µg of total RNA was employed to create complementary DNA (cDNA) following the manufacturer’s methodology (Thermo Fisher, United States). RT-PCR was carried out utilizing a PowerUp^TM^ SYBR^TM^ Green Master Mix kit utilizing a QuantStudio^TM^ Dx Real-Time PCR Device (Life Technologies Corporation, United States) (Thermo Fisher, United States). In brief, 40 rounds (95 ^°^C for 15 sec, 60 ^°^C for 15 sec , and 72 ^°^C for 1 min) were used. Glyceraldehyde 3-phosphate dehydrogenase (GAPDH) mRNA expression was employed as the standard solution for every specimen, and the proportional mRNA expression levels of the target genes were estimated utilizing proportional amounts. The profiles of the gene primers were as follows:

Sox11: F: CGAGCCTGTACGACGAAGTG

R: AAGCTCAGGTCGAACATGAGG

***Western blot***

Western blot was carried out as illustrated previously (21). Firstly, Radio Immunoprecipitation Assay (RIPA) lysis buffer (Beyotime, China) was used to homogenize the samples from peri-injury cortex tissues and then adjusted at 12,000 g for 20 min at 4 ^°^C. Bicinchoninic acid (BCA) was added to the supernatants, and protein density was detected with the Pierce^TM^ BCA Protein Assay Kit (Thermo Fisher, United States). The specimens (30 µg) were loaded to sodium 8% SDS-polyacrylamide gel (Beyotime, China) for electrophoresis and then transferred to polyvinylidene difluoride (PVDF) membranes (Millipore, United States). The following antibodies were applied to membranes with the use of blocking buffer (Beyotime, China) at room temperature for 1 hr and kept overnight at 4 ^°^C: rabbit Anti-Sox11 (1:2000, ab234996, Abcam, United States) and rabbit anti-Caspase-3 (1:500, ab13847, Abcam, United States). As a loading control, rabbit anti-GAPDH (1:10,000, G9545, Sigma, United States) was employed. Goat anti-rabbit IgG-HRP secondary antibody (1:10,000, 31460, Invitrogen, United States) was administered to the membranes for 1 hr at room temperature. The Immobilon^TM^ Western Chemiluminescent HRP Substrate (Millipore, United States) and a capturing technique (GE Healthcare Bio-Sciences, China) were applied to visualize the bands. Image J program (National Institutes of Health) was utilized to evaluate all findings.

***Immunofluorescence staining***

Double immunofluorescence staining was carried out as described previously (22). The sections were dewaxed and rinsed thrice in phosphate-buffered saline (PBS) after baking for 1 hr at 70 ^°^C. The sections were then supplemented with a blocking solution (Beyotime, China) for 60 min at room environment, then preserved at 4 ^°^C for 1 day with the main antibodies rabbit anti-Sox11 (1:200), mouse anti-NeuN (1:1000, ab104224, Abcam, United States), and mouse anti-CD11b (1:100; MCA275, Bio-Rad, United States). After that, the sections were treated for 60 min at room environment with both Alexa Fluor 488 donkey anti-rabbit IgG antibody (1:800, A-21206, Invitrogen, United States) and Alexa Fluor 555 donkey anti-mouse IgG antibody (1:800, A32773, Invitrogen, United States) as secondary antibodies. 4’,6-diamidino-2-phenylindole dihydrochloride (DAPI) anti-fluorescence quenching solution (YEASEN, China) was employed to encapsulate the sections before they could be examined by using a fluorescence microscope (OLYMPUS, U-RFL-T, Japan).

***Neurological score***

The adjusted Garcia examination was used to examine sensorimotor problems in a blinded manner at 72 hr after SBI (23, 24). The test was divided into seven parts, including action in the cage, body deep sensation, whisker touch reactions, corresponding limb movement, turning, overstretching of the forepaws, and climbing ability. Each variable recorded a score from 0 to 3, with a composite top score of 21. The higher the score, the better performance.

***Brain edema***

Employing a wet/dry method assessed the brain-edema index (25). To determine the wet weight, brain tissue was gathered and weighed. The cells were then dried for 72 hr at 100 ^°^C before recording the mass once more to define the dry mass. The proportion of water content of the brain was detected by the following equation: [(wet weight-dry weight)/wet weight] 100%.

***Staining of TUNEL***

It was employed to assess cell death based on the guidelines of the manufacturer (Abcam, United States). The sections were dewaxed in xylene, washed twice with PBS, and then supplemented with 20 µg/ml proteinase K for 20 min at 37 ^°^C. Three PBS rinses were performed. Then the segments were treated with TUNEL working solution at 37 ^°^C for 1 hr without any light. The segments were covered with DAPI anti-fluorescence quenching solution (YEASEN, China) and examined by using a fluorescence microscope after three PBS rinses (OLYMPUS, U-RFL-T, Japan).

***Fluoro-Jade C (FJC) staining***

FJC staining was employed to mark necrosis with a kit from Biosensis (USA). The sections were set in a 70 ^°^C oven for 1 hr and dewaxed in xylene, 100% ethanol, 80% alcohol, and 70% ethanol. The sections were rinsed two times with double-distilled H_2_0 and incubated with solution B (one part of potassium permanganate mixed with nine parts of distilled water) for 10 min. After two washes with double-distilled H_2_O, the sections were incubated with solution C (one part of FJC solution mixed with nine parts of distilled water) for 30 min in the dark and rinsed with distilled water. Then the sections were dried at 60 ^°^C for 10 min, followed by soaking in xylene for 1 min. Finally, sealing was carried out with Neutral Balsam (YEASEN) before observation under an OLYMPUS fluorescent microscope.

***Statistical analyses***

GraphPad Prism 8.0 software was employed to analyze all the results. All information was displayed as mean±standard deviation. The Kolmogorov-Smirnov test was employed to define the normality. An ANOVA with Dunnett’s *post-hoc* analysis was utilized to assess both RT-PCR results and WB findings. To analyze the results from the immunofluorescence staining, a student’s *t*-test was employed. In Experiment 2, cohort differences were analyzed utilizing a one-way ANOVA with Tukey’s post-hoc test. *P*<0.05 was selected as statistically significant.

## Results

***Expression of mRNA and protein levels of Sox11 in the brain after SBI***

**Figure 2:**
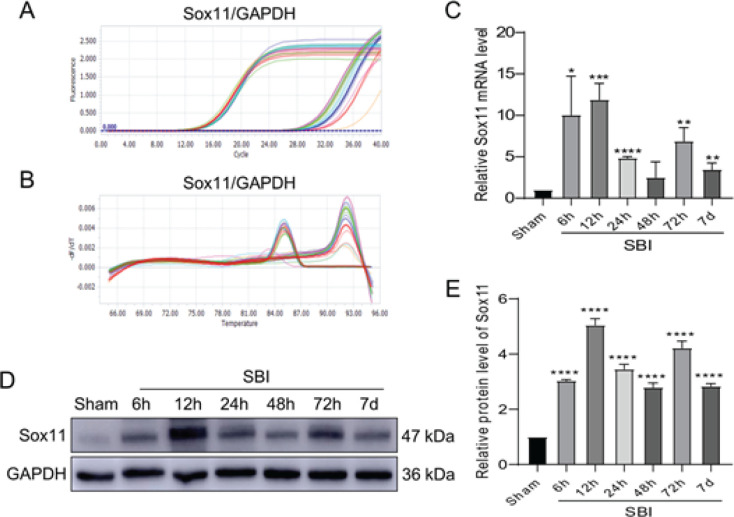
mRNA and protein expression of Sox11 in the peri-injury cortex following SBI in rats

RT-PCR and WB studies were used to determine the amount of endogenous Sox11 expression at 6 hr, 12 hr, 24 hr, 48 hr, 72 hr, and 7 d after SBI. The amplification plots and melting temperature curves for these genes revealed their cycle limits and that only one production per gene was produced (Figure 2A, 2B). Following SBI, the amount of Sox11 mRNA was first elevated, peaking at 12 hr. After this peak, the Sox11 mRNA level gradually decreased and reached the second peak at 72 hr, but decreased compared with that of 12 hr (Figure 2C). The WB results for protein levels of Sox11 showed the same results as RT-PCR (Figure 2D, 2E).

***Expression of Sox11 in peri-injury cortical cells following SBI***

**Figure 3:**
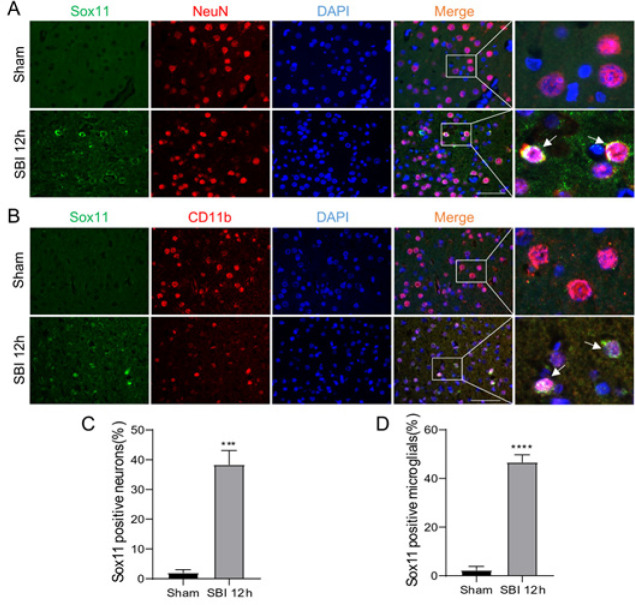
Location of Sox11 in the peri-injury cortex following SBI in rats

Immunofluorescence labeling was employed to determine the location of Sox11 in the brain 12 hr after SBI using the neuronal marker NeuN or the microglial marker CD11b. According to immunofluorescence studies, the SBI (12 hr) cohort had more Sox11-positive neurons (Figure 3A, 3C) and microglial cells (Figure 3B, 3D) than the Sham cohort, which was in line with the findings of WB. Thus, Sox11 may have contributed to the pathologic development of SBI.

***Impact of siRNA intervention on the protein expression of Sox11 following SBI***

**Figure 4:**
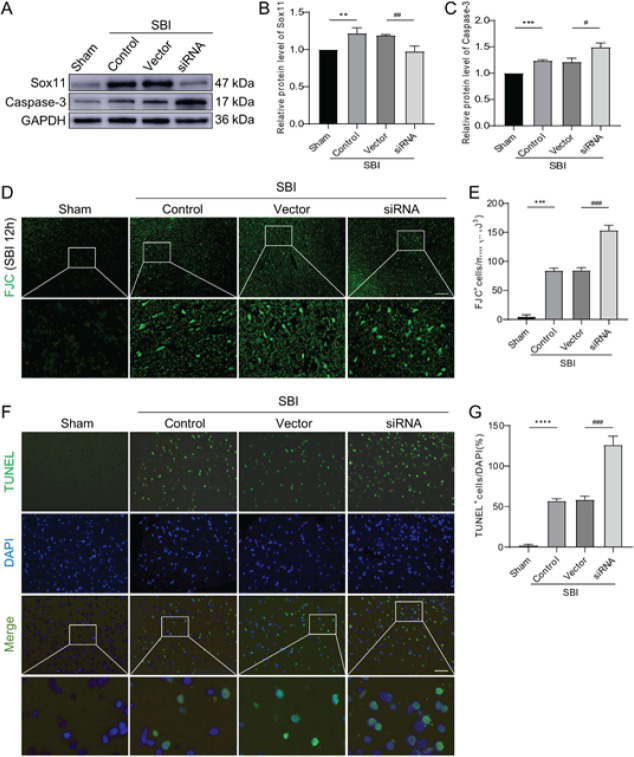
Impact of siRNA intervention after SBI in rats

There was a remarkable elevation in the protein quantity of Sox11 at 12 h following SBI in Experiment 1, compared with that in the Sham cohort. Therefore, in the following experiment, 12 hr was selected for the evaluation. During this experiment, as the expression of Sox11 was aimed to be reduced, the study cohort was treated with Sox11 siRNA while the control cohort was treated with a siRNA vector. The Sham cohort had a low protein expression level and the SBI and Vector cohorts significantly increased, respectively. As a result, a substantial variance between the SBI cohort and Sham cohort was recorded, with no differences between the SBI cohort and Vector cohort. The siRNA cohort revealed substantial variances from the Vector cohort after siRNA intervention (Figures 4A, 4B). The results indicate that the procedure to inhibit the expression of Sox11 is effective.

***Impact of siRNA intervention on neuronal degeneration and brain cell death following SBI***

Aiming to detect the function of Sox11 in neuronal cell death, WB of Caspase-3 and TUNEL staining, as well as FJC staining, were performed. From WB, we identified that the expression of Caspase-3 in the siRNA cohort was elevated when compared with the Vector cohort. The SBI cohort was substantially different from the Sham cohort and not different from the vector cohort (Figure 4A, 4C). To examine the function of Sox11 in SBI-induced apoptosis and necrosis, TUNEL and FJC staining of the damaged peripheral cortex was performed 12 hr after SBI. Under Sox11 deficiency, there was a substantial elevation in the amount of apoptosis (Figure 4F, 4G) and necrosis (Figure 4D, 4E). From these results, cell death could be aggravated by Sox11 deficiency.

***Neurological behavior scores in SBI rat after siRNA intervention***

**Figure 5:**
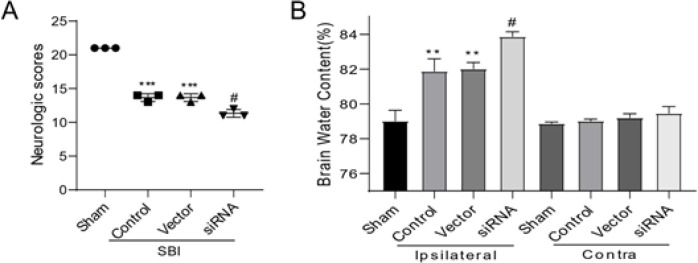
Neurological behavior scores and water content of the brain following surgical brain injury (SBI) in rats

The observed neurological behavior scores were experimented with the adjusted Garcia test. The SBI cohort is substantially different from the Sham cohort. The SBI cohort showed no differences from the Vector cohort. The siRNA cohort revealed substantial differences from the Vector cohort (Figure 5A). During SBI, the increase in neurological behavior impairment can be promoted by the inhibition of the Sox11 expression level.

***Cerebral edema index in SBI rats supplemented with siRNA injection***

The brain water content of SBI, Vector, and siRNA cohorts after SBI was significantly elevated when comparing the Sham cohort. Furthermore, the brain water level of the SBI cohort was substantially larger than that of the Sham cohort. Additionally, after the siRNA interference at 12 hr following SBI, the brain water level in the damaged hemispheres increased. Furthermore, no change in brain edema was observed in the hemisphere opposite the damage (Figure 5B).

(A) Brain tissue from the peri-injury in the SBI cohort and the identical site in the Sham cohort in rats was collected for the test. The shadow portion is the experimental detection area. (B) Experiment 1 was created to detect the expression levels and sites of Sox11 over time following SBI in rats and indicate the appropriate time point for the next experiment. (C) Experiment 2 was created to identify the impacts of Sox11 on early brain damage following SBI in rats

SBI: Surgical brain injury; SOX11: sex-determining regions-Y box 11

(A, B, C) Proportional mRNA expression levels of Sox11 were examined utilizing the proportion of the number of target mRNAs to the GAPDH mRNA. (D, E) Western blot was carried out to define the protein levels of Sox11 in the Sham and SBI cohorts at 6 hr, 12 hr, 24 hr, 48 hr, 72 hr, and 7 d. Each protein’s relative concentrations were normalized to the Sham cohort. Statistical analyses were carried out by utilizing one-way ANOVA followed by Dunnett’s *post hoc* test. Data are expressed as the mean±SD. n=6 for each group. *P<0.05, **P<0.01, ***P<0.001, ****P<0.0001 vs Sham

SBI: Surgical brain injury; SOX11: sex-determining regions-Y box 11

(A, B) Representative double-immunofluorescence staining images of Sox11 (green) with NeuN /CD11b (red)-marked cells to illustrate expression profiles in the Sham and 12 hr SBI groups. DAPI (blue) was employed to stain the nuclei fluorescently. The arrows indicate the colocalization of Sox11 with neurons/microglia. (C, D) Density of Sox11+ cells. Scale bar=50 µm. Data are expressed as the mean±SD. n=6 for each cohort. Statistical evaluation was performed using Student’s t-test. ***P<0.001, ****P<0.0001 vs Sham

SBI: Surgical brain injury; SOX11: sex-determining regions-Y box 11; DAPI: 4,6-diamino-2-phenyl indole

(A, B, C) Protein expression levels of Sox11 and Caspase-3 were carried out to evaluate the value of siRNA intervention in the peri-injury cortex at 12 hr following SBI. (D, F) Coronal sections of the cerebral cortex from the Sham group, Control cohort, Vector cohort, and siRNA cohort were stained for FJC (green), TUNEL (green), and DAPI (blue). (E, G) Numbers of FJC+ or TUNEL+ cells in the cerebral cortex after SBI. Scale bars=100 µm. Data are expressed as the mean±SD. n=6 for each group. One-way ANOVA was followed by Tukey’s *post hoc* test. **P<0.01, ***P<0.001, ****P<0.0001 vs Sham; ^#^*P<*0.05; ^##^*P<*0.01, ^###^*P<*0.001 vs Vector group

SBI: Surgical brain injury; SOX11: sex-determining regions-Y box 11; DAPI: 4,6-diamino-2-phenyl indole; FJC: Fluoro-Jade C

(A) Neurological behavior scores in SBI rats after Intervention of siRNA. (B) Quantitative analysis of brain water content in different cohorts in a dry/wet manner. Data are illustrated as the mean±SD, n=3 for each group. One-way ANOVA was followed by Tukey’s *post hoc* test. **P<0.01, ***P<0.001 vs Sham; ^#^*P<*0.05 vs Vector group

**Figure 6:**
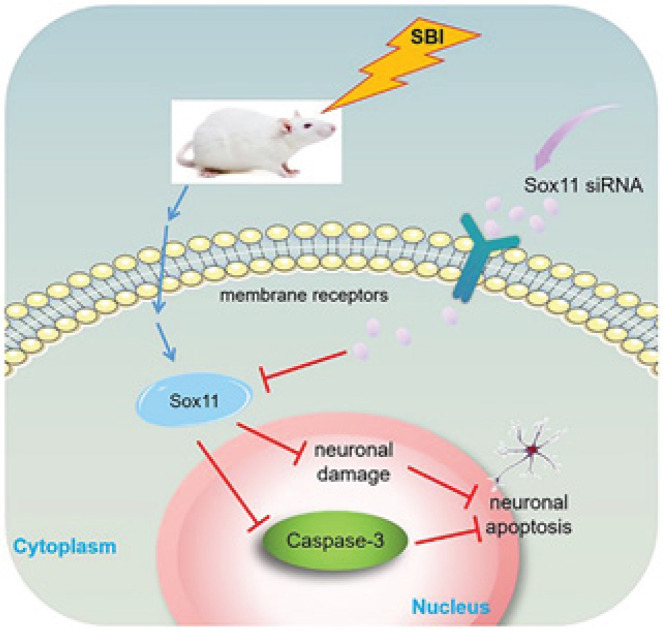
Schematic diagram showing the role of Sox11 following surgical brain injury (SBI) in rats

After SBI, the expression of Sox11 was increased, which influenced secondary brain injury by affecting the level of Caspase-3. Inhibition of Sox11 with siRNA may promote the transcription of Caspase-3, leading to neuronal injury and finally causing cell death, aggravating secondary brain injury after SBI

## Discussion

In this study, Sox11 is activated in the surrounding operation area after SBI by establishing the SBI right frontal lobe resection rat model. In Experiment 1, the Sox11 value in cortical tissues of the surgical area and neurons increased substantially after SBI and achieved its peak at 12 hr; it rose to the second peak at 72 hr and then declined to the same level as the Sham cohort at day 7 (Figure 2). In Experiment 2, we suppressed the expression of the Sox11 gene with siRNA. The treatment with Sox11 siRNA increased neuronal death in the surgical area, exacerbated brain edema, and aggravated neurological damage after SBI (Figure 4). Therefore, the lack of Sox11 contributed to an exacerbation of secondary brain injury after SBI, affected the level of cell death-related protein Caspase-3, and ultimately affected the survival of neurons. The schematic model is shown in Figure 6. 

Sox11 plays various interesting roles in development, bone remodeling, cancer, adult neurogenesis, and nerve regeneration. In normal healthy individuals, Sox11 expression is usually reduced while its control is stable. Sox genes are changed during brain damage and associated with pathophysiological processes such as neuroinflammation and cell death. Sox11 in the cerebral cortex was significantly induced following transient forebrain ischemia (15). Our study indicated that aggravated nerve injury after SBI induced increased expression of Sox11. The knockdown of Sox11 level influences neuroinflammation and the death of cells in various neurological diseases (26). When we knocked down Sox11 by administering siRNA via intracerebroventricular injection, the brain damage induced by SBI worsened. In the pathological background, Sox11 existed in different cellular compartments (27). Sox11 can be localized in the nucleus and cytoplasm during neurogenesis in embryos and adults (28, 29). In our study, Sox11 is mainly presented in the cytoplasm of the region around the damaged cortex (Figure 3). 

A substantial increase in apoptosis can occur in the absence of one or more SoxC proteins (5, 30). In addition, Sox11 is a candidate Caspase-6 interactor (31) and can reduce the activity of Caspase-3 (32). In our work, Sox11 knock-down exacerbated SBI-induced brain injury and increased Caspase-3 action, which revealed that deficiency of Sox11 aggravated neuronal apoptosis in SBI (Figure 4). Therefore, the findings reinforce the potential usage of Sox11 in brain injury treatment. We suggested that Sox11 was activated after SBI to protect neuronal cells. On the contrary, the intervention of siRNA raised the level of apoptosis-related protein Caspase-3, leading to neuronal cell death. 

However, the mechanism of Sox11 regulating neuronal apoptosis and the role of the cerebral cortex is still uncertain. Sox11 can control axon growth and its overexpression is critical for supporting axon development (33). A recent study of nerve injury has shown that Sox11 supports nerve growth by activating Sprr1a, a gene associated with regeneration (34). The same findings have been demonstrated in spinal cord traumatic injury, where over-expression of Sox11 contributed to the regeneration of neuronal cells after SCI, reduced the death of neuronal cells, and enhanced motion function affection in a spinal cord trauma mice model (35). Sox11 can regulate the transcription of Brain-derived neurotrophic factor (BDNF) in peripheral nerves (36). However, whether Sox11 can also regulate BDNF in brain injury has not been reported. It is worth noting that our study only explored the expression and localization of Sox11 after SBI, and its effects on nerve apoptosis and necrosis after secondary brain injury. The pathophysiological pathway of Sox11 on secondary brain injury was not discussed in detail. In the following experiments, we will study the pathophysiological mechanisms of Sox11 for secondary brain injury after SBI.

## Conclusion

Inhibiting Sox11 with siRNA may aggravate secondary brain injury after SBI by affecting the level of cell death-related protein Caspase-3. The results of this study provide a potential new treatment target for the creation of new medications, and practical management for the protection of damaged brain tissue is herewith proposed.

## Authors’ Contributions

J T and B D designed the experiments; J T, M W, J S, and L J performed experiments and collected data; J T, M W, and L C discussed the results and strategy; L C and B D supervised, directed, and managed the study; J T, M W, J S, L J, L C, and B D approved the final version to be published.

## Conflicts of Interest

The authors declare that they have no competing interests.
